# Periorbital Necrotizing Fasciitis: Case Presentation

**DOI:** 10.2196/52507

**Published:** 2023-11-28

**Authors:** Ryan S Huang, Nikhil S Patil, Yasser Khan

**Affiliations:** 1 Temerty Faculty of Medicine University of Toronto Toronto, ON Canada; 2 Michael DeGroote School of Medicine McMaster University Hamilton, ON Canada; 3 Department of Ophthalmology McMaster University Hamilton, ON Canada

**Keywords:** periorbital necrotizing fasciitis, Streptococcus pyogenes A, skin infection, soft tissue infection, dermatology infection, skin reaction, periorbital, necrotizing fasciitis, necrotizing, necrosis, case report, case reports, fasciitis, fatal, life-threatening, fascia, soft tissue, infection, pathology, pathophysiology, periorbital, eye, orbital, orbit, muscle, bacteria, bacterial, Streptococcus, inflammation, tissue, tissues

## Abstract

Necrotizing fasciitis (NF) is an aggressive and potentially life-threatening infection of the superficial fascia and surrounding skin, fat, fascia, muscle, and other soft tissue structures. Here, we outline the rare case of a 26-year-old man with a periorbital Streptococcus pyogenes A NF infection. Our case report underscores a unique instance of periorbital NF, distinctively presenting without any predisposing risk factors, shedding light on its presentation, treatment, and pathophysiology.

## Introduction

Necrotizing fasciitis (NF) or necrotizing soft tissue infection is a rare and severe infection of the skin, muscle, subcutaneous tissue, and underlying fascia. In the United States, approximately 0.4 in every 100,000 people per year are affected by NF, while it is as common as 1 in every 100,000 people in other countries [1]. NF is characterized by rapidly progressing soft tissue necrosis invading the superficial and deep fascia, spreading along the fascial plane, and if not treated immediately, it eventually spreads into the systemic circulation [2]. The infection is most commonly associated with trauma involving a laceration that introduces bacteria into the wound site [3,4]. The infectious agents for NF are variable, but it is most commonly a result of a group A β-hemolytic streptococcal (*Streptococcus pyogenes*) infection [5]. NF is a rare condition, especially in urban settings, and most often occurs in the abdomen, perineum, arms, or legs. It is exceedingly rare in the head and neck regions; however, NF is known to cross anatomical planes [3,4]. Periorbital NF infections are generally localized to the eyelid and penetrate from the skin to the tarsus plate fascia. It can be identified with specific features such as necrosis with purulent discharge, blistering, and nonspecific features such as localized tenderness, severe pain, and erythema at the site of infection [5]. The spread of infection beyond the eyelid to the eyebrow, down the cheek, or to the nose is a major indicator of severe NF. If left untreated, periorbital NF complications can lead to blindness, neuralgias, and even death [5]. To the best of our knowledge, there are few cases documenting the successful treatment of periorbital NF in the literature. Here, we present the successful treatment of a unique case of a young patient with periorbital NF without any preexisting risk factors.

### Case Presentation

**Figure 1:**
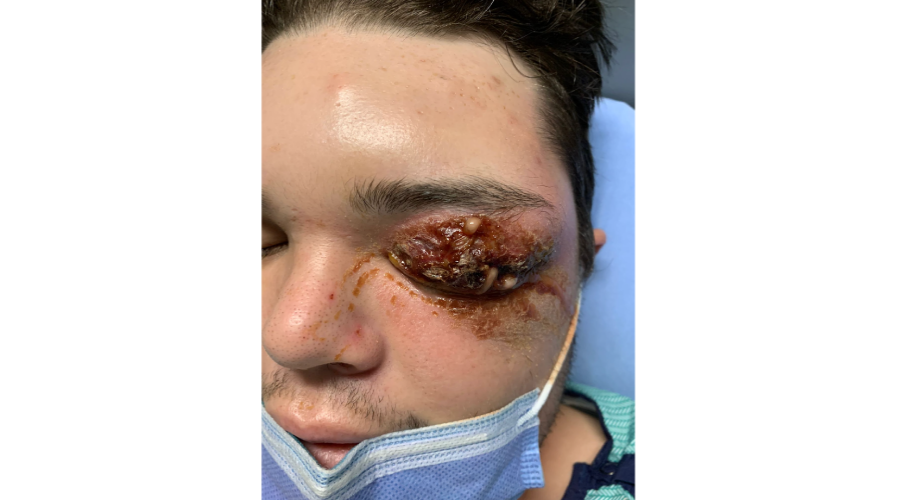
Patient presentation. The patient had left eye (OS) swelling with difficulty opening the eye and pain. Preseptal purulent discharge and tissue emphysema were observed.

**Figure 2:**
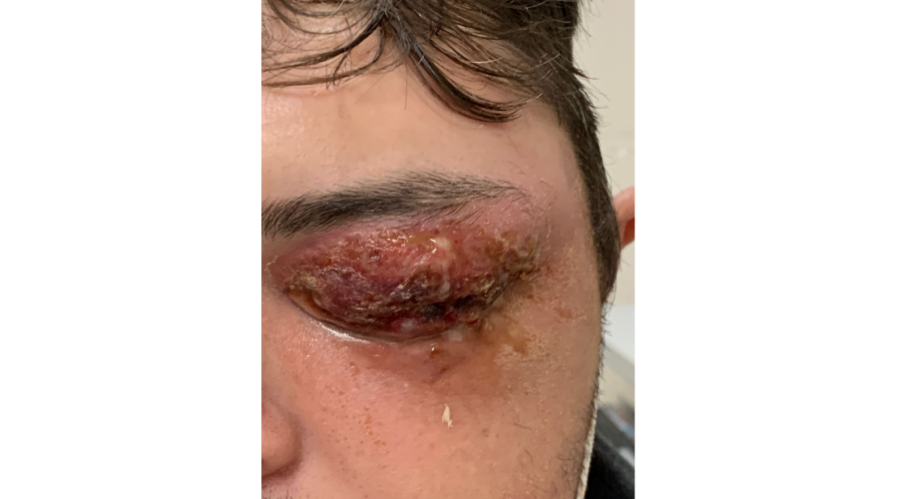
Posttreatment day 1. The patient improved the following day after treatment with intravenous Tazocin and vancomycin.

A 26-year-old male patient was referred to us for left upper lid periorbital bruising after a bicycle fall onto asphalt 3 days prior. He had no previous medical history or risk factors for NF such as intravenous drug use, immunosuppression medications, advanced age, obesity, or malnutrition. The patient had left eye (OS) swelling with discharge, difficulty opening the eye, and moderate to severe pain. Preseptal purulent discharge and tissue emphysema were observed (Figure 1). Inflammatory changes extended to the left temporalis, masseter, and buccinator muscles, along with enlargement of the left parotid gland and intraparotid lymph nodes. An orbital computed tomography demonstrated extensive preseptal cellulitis, along with preorbital gas and fluid accumulation. The discharge was drained and cultured, revealing a *S pyogenes* A infection. The patient was treated with intravenous Tazocin and vancomycin immediately, leading to a decrease in inflammation over the following day (Figure 2). He was scheduled for emergency surgical debridement. During the procedure, the oculoplastics team found extensive necrosis of the left upper lid skin, orbicularis oculi, levator palpebrae superioris muscles, tarsus, surrounding fat, and neurovasculature. No necrosis was seen past the septum. After the debridement, the tissue was washed with Proviodine and treated with antibiotics. The tissue was then packed and dressed using Tobradex and bacitracin-soaked gauze. After 3 days of the surgery, the patient had no pain, erythema, ulcerations, or necrotic tissue. Granulation tissue had started to develop, but there was still a considerable amount of swelling restricting palpebral movement. At 1-week follow-up, the swelling had improved substantially, and the function of the levator palpebrae superioris and orbicularis oculi was regained.

### Ethical Considerations

Informed consent was obtained, in writing, from the patient for this publication.

## Discussion

Our case report presents the successful treatment of a rare case of periorbital NF in a patient with no risk factors, manifesting as extensive preseptal cellulitis, along with preorbital gas and fluid accumulation. Patients with an NF infection often have predisposing factors including diabetes mellitus, peripheral vascular disease, chronic alcoholism, malignancy, or immunosuppression [6]. Periorbital NF can be a result of a variety of pathogens including *Staphylococcus aureus*, *Cryptococcus neoformans*, *Pseudomonas aeruginosa*, and *Clostridium perfringens*. The most common pathogenic infection is *S pyogenes* A. NF can present in 1 of 2 types: type I or type II. Type I NF is a polymicrobial infection, with both aerobic and anaerobic bacteria, enterobacterium, and gram-negative bacilli. Type II NF is a result of a streptococcal infection and is the most prevalent causative agent of periorbital NF [6].

The pathogenesis of the disease is mediated by bacterial toxins, such as M protein and hyaluronic acid capsules from *S pyogenes*, causing the evasion of leukocyte phagocytosis and humoral immune surveillance [7]. Other toxins are responsible for toxin-induced intravascular platelet aggregation, preventing leukocytes from reaching the site of infection, which in turn creates a hypoxic state decreasing polymorphonuclear neutrophil function. While the bacterial toxins traverse the fascia, they induce platelet aggregation to thrombose vessels supplying the skin, allowing the toxin-induced necrosis to proliferate. As a result of the dermal hypoxic conditions, hemorrhagic bulla and a purple hue form. It is important to note that preseptal infections have an uncompromised septum. Therefore, only superficial structures and a moderate debridement are needed to remove the necrosis with most of the orbicularis intact, as shown in our case. However, in postseptal infections, the septum is compromised and therefore the infection can spread into the orbit and can result in proptosis. This can lead to postseptal disease where the infection can spread through the optic nerve to the brain and debridement may not be enough, requiring enucleation of the eye.

While our case report primarily focused on the successful treatment and manifestations of periorbital NF, the long-term prognosis and implications for patients deserve emphasis. In the absence of risk factors, it is paramount for clinicians to maintain a high index of suspicion for NF, especially given its rarity and potentially rapid progression. Patients without risk factors can present with more subtle and atypical symptoms, delaying diagnosis, and potentially worsening outcomes. The long-term implications for such patients are multifaceted. On a physiological level, even after successful treatment, these patients might be at risk for residual scarring, functional impairment, and potential cosmetic concerns. Moreover, due to the unexpected nature of the disease in these individuals, there might be psychological implications, including anxiety and concerns about recurrence. Regular follow-ups with these patients are crucial, not only to monitor and manage potential complications but also to provide emotional and psychological support. Early rehabilitation, counseling, and patient education can play a pivotal role in optimizing their quality-of-life after treatment. In conclusion, the success of our described treatment approach not only underscores its viability for similar cases but also sets a precedent for potential therapeutic strategies in managing periorbital NF. Future research focusing on novel biomarkers for NF may offer insights into prevention and early detection strategies, especially in patients without conventional risk factors.

## Abbreviations

NF: necrotizing fasciitis
OS: left eye

## Data Availability

The data supporting the findings of this study are available on request from the corresponding author, YK. The data are not publicly available to protect the privacy of research participants.

## References

[1] Khalid M, Junejo S, Mir F (2018). Invasive community acquired methicillin-resistant staphylococcal aureus (CA-MRSA) infections in children. J Coll Physicians Surg Pak.

[2] Shindo ML, Nalbone VP, Dougherty WR (1997). Necrotizing fasciitis of the face. Laryngoscope.

[3] Bisno AL, Stevens DL (1996). Streptococcal infections of skin and soft tissues. N Engl J Med.

[4] Kronish JW, McLeish WM (1991). Eyelid necrosis and periorbital necrotizing fasciitis. report of a case and review of the literature. Ophthalmology.

[5] Lazzeri D, Lazzeri S, Figus M, Tascini C, Bocci G, Colizzi L, Giannotti G, Lorenzetti F, Gandini D, Danesi R, Menichetti F, Del Tacca M, Nardi M, Pantaloni M (2010). Periorbital necrotising fasciitis. Br J Ophthalmol.

[6] Tessier JM, Sanders J, Sartelli M, Ulrych J, De Simone B, Grabowski J, Buckman S, Duane TM (2020). Necrotizing soft tissue infections: a focused review of pathophysiology, diagnosis, operative management, antimicrobial therapy, and pediatrics. Surg Infect (Larchmt).

[7] Georgiou A, Haque SA, Henderson H, Woollard A (2021). Necrotizing fasciitis of the periorbital region: from presentation to reconstructive journey. Eur J Plast Surg.

