# Correction: Periorbital Necrotizing Fasciitis: Case Presentation

**DOI:** 10.2196/108273

**Published:** 2026-08-18

**Authors:** JMIR Publications Editorial Office

**Affiliations:** 1 JMIR Publications Toronto, ON Canada

Following the publication of “Periorbital Necrotizing Fasciitis: Case Presentation” [1], concerns were raised regarding a potential conflict of interest between reviewer Karen Wong Riff and the authors. This concern included shared affiliation with members of the author list. This conflict was not disclosed to the JMIR Publications Editorial Office as per journal policy.

This article has been subsequently reassessed by the editor in chief, who determined that the content of this review did not unduly influence the decision to accept the article. The decision to accept the submission was based primarily on the remaining reviewers’ comments and the editor in chief’s contemporary appraisal of the manuscript.

As such, the JMIR Publications Editorial Office issues this corrigendum to update the Conflicts of Interest statement:

Reviewer Karen Wong Riff was found to have an undisclosed conflict of interest with members of the author list under the terms of JMIR Publications policy. The editor in chief determined that the content of the review did not unduly influence the decision to accept the article. The decision to accept the submission was based primarily on the remaining reviewers’ comments and the editor in chief’s contemporary appraisal of the manuscript.

The JMIR Publications Editorial Office further issues an additional corrigendum to update the informed consent information, which is included in the "Ethical Considerations" subsection as follows:

*Informed consent was obtained, in writing, from the patient for this publication.*

We regret that these issues were not identified before publication.

The correction will appear in the online version of the paper on the JMIR Publications website together with the publication of this correction notice. Because this was made after submission to PubMed, PubMed Central, and other full-text repositories, the corrected article has also been resubmitted to those repositories.
